# Mesenchymal Stromal/Stem Cell-Based Therapies for Liver Regeneration: Current Status and Future Directions

**DOI:** 10.3390/ijms27020619

**Published:** 2026-01-07

**Authors:** Seohyun Choi, Jaemin Jeong

**Affiliations:** Department of Biohealth Convergence, College of Science and Convergence Technology, Seoul Women’s University, Seoul 01797, Republic of Korea; cswind25@gmail.com

**Keywords:** mesenchymal stem cells, liver regeneration, immunomodulation, fibrosis, extracellular vesicles, exosomes, cell therapy, hepatology

## Abstract

The global burden of acute and chronic liver diseases warrants safe and effective regenerative therapies that can complement or defer liver transplantation. Mesenchymal stromal/stem cells (MSCs) have been recognized as versatile biologics that modulate inflammation, reverse fibrosis, and promote hepatic repair predominantly through paracrine signaling. In hepatic milieu, MSCs act on hepatocytes, hepatic stellate cells, endothelial cells, and immune cell subsets through trophic factors and extracellular vesicles (EVs). Despite demonstrating hepatocyte-like differentiation of MSCs, their in vivo efficacy is primarily attributed to micro-environmental reprogramming rather than durable engraftment. This review covers MSC biology, liver regeneration, and cell-based versus EV therapies, including administration, dosing, quality, and safety. Future directions focus on biomarkers, multi-center trials, and engineered MSC/EV platforms for scalable personalized liver regeneration.

## 1. Introduction

Liver diseases represent a substantial global health burden, accounting for approximately 4% of all deaths worldwide and an estimated 1.5 million deaths each year [1]. Recent global epidemiological analyses estimate that chronic liver disease remains a significant cause of morbidity and mortality, with an estimated 58.4 million cases of liver cirrhosis recorded worldwide in 2021 as reported in recent analyses [1]. Major contributors to liver disease–related mortality include cirrhosis associated with chronic hepatitis B and C infection, alcohol-associated liver disease, metabolic dysfunction-associated steatotic liver disease (MASLD), and hepatocellular carcinoma [2,3]. Acute liver injury (ALI), induced mainly by viral infection and less frequently by drug toxicity, accounts for a smaller proportion of cases [2].

Among organs, the liver possesses a remarkable regenerative capacity and can restore its mass following hepatectomy. However, chronic injury leads to predominant inflammation, fibrosis, and vascular abnormalities rather than regeneration and recovery [2]. The injury progresses towards fibrosis, creating a fibrotic environment [2].

Orthotopic liver transplantation is the only radical treatment for end-stage liver disease. However, its widespread application is limited by its high cost, shortage of donors, and the need for lifelong immunosuppressive therapy [4,5]. Moreover, complications related to immunosuppression, such as renal dysfunction, metabolic syndrome, cardiovascular disease, de novo malignancies, infections, and osteoporosis, as well as organ rejection, compromise long-term success. These factors also lead to poor quality of life and increased mortality after transplantation [6].

In light of these limitations, regenerative therapies that modulate the hepatic microenvironment have emerged as a promising strategy to restore functional interactions among hepatocytes, nonparenchymal cells, and the extracellular matrix (ECM), while improving metabolic and inflammatory balance [7]. Among the various regenerative strategies under investigation, mesenchymal stromal/stem cells (MSCs) and MSC-derived exosomes have attracted attention because they offer diverse regeneration-promoting mechanisms [7].

The most salient advantage of MSCs is their low immunogenicity. They can inhibit excessive immune responses during liver injury, participate in preventing or reversing fibrosis progression, and promote adequate blood supply by stimulating angiogenic factors [8]. MSC secretomes, comprising soluble components such as growth factors, cytokines, and chemokines and insoluble components such as extracellular vesicles (EVs), regulate immune cell activity, modulate anti-inflammatory and anti-apoptotic responses, and promote angiogenesis [9]. Together, these properties support liver regeneration.

The multifunctionality of MSCs provides a biological rationale for testing MSC-based strategies for the treatment of liver diseases, such as acute injury, acute-on-chronic liver failure, and cirrhosis [10,11,12]. However, because of the variation in MSC sources, culture conditions, dosing strategies, and delivery routes, standardized protocols and clinically relevant assays are needed to ensure consistent and reliable therapeutic effects [13]. While previous reviews have often focused separately on either preclinical mechanisms or clinical outcomes, this review integrates mechanistic insights, preclinical and clinical evidence, manufacturing, quality control, and regulatory considerations, offering a comprehensive and forward-looking perspective on MSC- and EV-based liver regenerative therapies.

## 2. MSC Biology and Source-Dependent Properties

### 2.1. Cellular Characteristics of MSCs

Although the terms “mesenchymal stromal cells” and “mesenchymal stem cells” (both abbreviated as MSCs) are often used interchangeably in the literature, the International Society for Cellular Therapy has provided a formal definition specifically for mesenchymal stromal cells. According to this definition, mesenchymal stromal cells must (i) adhere to plastic under standard culture conditions; (ii) express specific surface markers—positive expression for CD73, CD90, and CD105 and negative expression for hematopoietic markers such as CD34, CD45, CD14, CD19, and HLA-DR (although some variation may exist depending on tissue source and culture conditions); and (iii) demonstrate in vitro trilineage differentiation potential into osteoblasts, adipocytes, and chondrocytes [14].

By contrast, the use of the term mesenchymal stem cells requires clear evidence of in vivo self-renewal and multipotency [14]—criteria that are not routinely demonstrated in most studies. However, since many studies use these terms inconsistently and often without verifying true stemness, we will refer to them collectively as MSCs throughout this review for the sake of consistency.

Initially isolated from bone marrow, MSCs have since been identified in various adult and perinatal tissues, including adipose tissue, umbilical cord, placenta, dental pulp, and synovial membrane [15,16,17]. MSCs exhibit considerable heterogeneity depending on their tissue source, donor age and health status, and ex vivo culture conditions, which remains a major barrier to therapeutic standardization [18,19,20].

### 2.2. Source-Dependent Properties

MSCs can be obtained from multiple adult and perinatal tissues, and their tissue of origin influences their proliferative capacity, secretome composition, and immunologic properties. The first MSCs isolated from bone marrow demonstrated strong osteogenic potential and immunomodulatory effects [21]. Subcutaneous or muscular transplantation of these bone marrow-derived MSCs resulted in the development of reticular tissue and bone formation through M2 macrophage polarization and osteoblast activation [21,22]. However, the isolation of these MSCs requires an invasive aspiration technique, and their percentage in bone marrow, at approximately 0.001–0.01%, is extremely small, which limits their availability [23].

Adipose-derived MSCs can be easily obtained in high numbers from liposuction aspirates, yielding approximately 4 × 10^5^ cells/mL [24]. They secrete proteins, cytokines, growth factors, noncoding RNAs, microRNAs, and EVs. Adipose-derived MSCs promote angiogenesis, proliferation, and migration of keratinocytes and fibroblasts; induce M2 polarization of macrophages; and contribute to extracellular remodeling. The efficient proliferation of these MSCs in culture and their secretion of a potent pro-regenerative secretome make them well-suited for scalable manufacturing and repeated collection [25].

Perinatal MSCs, including Wharton’s jelly-derived MSCs (WJ-MSCs) [26], umbilical cord blood–derived MSCs (UC-MSCs) [27], and placenta-derived MSCs (PL-MSCs) [28], exhibit lower immunogenicity and greater proliferation potential. This is reflected by the low or absent expression of co-stimulatory molecules (CD40, CD80, and CD86) and major histocompatibility class II antigens (HLA-DQ, DP, DR) [26,27,28]. PL-MSCs also express higher levels of OCT4 and SOX2, suggesting a more primitive phenotype [28], and show a higher proliferation rate with a doubling time of approximately 41 h [28]. UC-MSCs typically have a doubling time of approximately 68 h, and this may vary with maternal age [29]. Perinatal MSCs exert antifibrotic and anti-inflammatory effects. In an in vitro skeletal muscle fibrosis model, WJ-MSCs inhibited fibrosis in myotubes by secreting matrix metalloproteinase-1 (MMP-1) [30]. In a dextran sulfate sodium–induced intestinal fibrosis model, conditioned media from UC-MSCs/PL-MSCs suppressed excessive ECM accumulation and myofibroblast activation by inhibiting the transforming growth factor-β (TGF-β)/Smad and RhoA/myocardin-related transcription factor/serum response factor signaling pathways [31]. Furthermore, in a bleomycin-induced lung fibrosis model, injection of WJ-MSCs downregulated the expression of pro-fibrotic and proinflammatory cytokines, including TGF-β, interferon-γ (IFN- γ), tumor necrosis factor-α (TNF- α), and macrophage migration inhibitory factor, while promoting ECM degradation through the upregulation of MMP-2 expression [32].

MSC-like cells can be generated from induced pluripotent stem cells (iPSCs). These cells closely resemble native MSCs in terms of surface markers such as CD73, CD90, CD105, CD146, and CD166, while lacking iPSC markers such as TRA-1-60 and TRA-1-81 [33,34]. The resulting iPSC-derived MSCs offer the advantage of long-term expansion, reportedly up to 120 passages [33]. Although precise quantitative data from recent clinical-grade iMSC lines are limited, preclinical studies suggest that chromosomal abnormalities can occur in a small fraction of expanded lines, raising concerns regarding genomic stability and potential tumorigenic risk and underscoring the need for rigorous genomic screening and quality control [33,34,35]. Additionally, challenges such as potential heterogeneity, incomplete in vivo functionality, and complexity of manufacturing remain important considerations for clinical translation [33,34,35]. The source-dependent properties of MSCs relevant for hepatic applications are summarized in Table 1.

### 2.3. Influence of Culture Conditions on Potency

Culture conditions strongly influence the functional capacity of MSCs by altering their gene expression, epigenetic profiles, and secretion patterns. Four main culture conditions have been found to exert a profound influence. First, under hypoxic conditions that mimic the physiological niche, MSCs increase the secretion of several growth factors, including vascular endothelial growth factor (VEGF), hepatocyte growth factor (HGF), and basic fibroblast growth factor. These conditions also support MSC self-renewal through hypoxia-inducible factor (HIF)-dependent mechanisms [36]. Meanwhile, their colony-forming ability is enhanced independent of HIF [36]. Moreover, under hypoxia, mitochondria export acetyl-CoA to the cytosol and nucleus, enabling sufficient histone acetylation, which keeps chromatin in a more open and accessible state, thereby preserving the differentiation potential of MSCs [37].

Second, exposure to proinflammatory cytokines modulates the functional capacity of MSCs. Exposing MSCs to IFN-γ and TNF-α resulted in the upregulation of cyclooxygenase-2 and prostaglandin E2 (PGE2), while exposure to IFN-γ induced the expression of programmed death-ligand 1 and indoleamine 2,3-dioxygenase, resulting in enhanced suppression of alloantigen-driven proliferation [38,39]. Furthermore, IFN-γ and TNF-α enhanced the expression of CD73 and CD5L in MSC-derived EVs (MSC-EVs), contributing to their immunosuppressive properties and the induction of macrophage M2 polarization, respectively [40].

Third, spheroid culture conditions create a mildly hypoxic environment, which maintains pluripotency and self-renewal capacity via HIF-1α and HIF-2α [41]. Compared with 2D–cultured MSCs, MSC spheroids exhibit stronger Toll-like receptor 3 activation, resulting in enhanced anti-inflammatory activity through the increased secretion of interleukin-10 (IL-10) and PGE2 [42]. MSC spheroids also increase the expression of antifibrotic and hepatoprotective factors, such as HGF, stromal cell-derived factor-1, and MMPs, leading to a greater reduction in collagen deposition, improved liver function, and decreased expression of fibrotic markers [43]. In addition, MSC spheroids exhibit proangiogenic effects by increasing the secretion of proangiogenic microRNAs, such as miR-21-5p, miR-126-5p, and miR-130a-3p [44].

Fourth, transitioning to xeno- and serum-free culture conditions results in significantly greater expansion and prevents triggering immune responses or allergic reactions, which is essential for clinical applications and compliance with good manufacturing practice (GMP) standards [45,46]. The major culture variables and their functional consequences on MSC potency are summarized in Table 2.

## 3. Mechanisms of Action in Liver Regeneration

**Figure 1:**
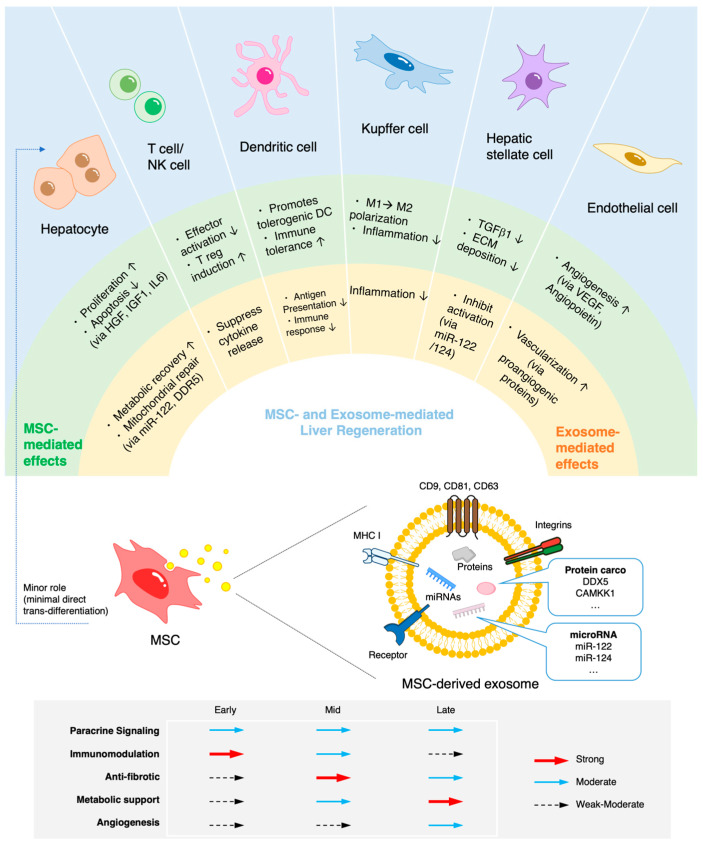
Mechanisms of MSC-mediated liver regeneration. MSC effects do not occur strictly sequentially; rather, they represent overlapping processes with variable predominance depending on the phase of liver injury and repair. MSCs promote liver regeneration through paracrine signaling, immunomodulation, antifibrotic activity, metabolic support, and angiogenesis. They secrete growth factors such as HGF, IGF-1, and IL-6 to enhance hepatocyte proliferation and survival; modulate immune responses by suppressing effector T and NK cells and promoting regulatory T cells and M2 macrophages; inhibit fibrosis by downregulating TGF-β1 and normalizing the ECM; improve tissue perfusion through angiogenic factors such as VEGF and angiopoietin; and restore hepatocyte metabolism via mitochondrial transfer and extracellular vesicles carrying functional RNAs and proteins. MSC trans-differentiation into hepatocyte-like cells is negligible. Arrows shown in the lower panel indicate the relative strength of each MSC- and EV-mediated effect across early, mid, and late phases of liver regeneration, highlighting overlapping contributions rather than strict sequential order. MSC, mesenchymal stromal/stem cell; HGF, hepatocyte growth factor; IGF1, insulin-like growth factor 1; IL6, interleukin-6; NK cell, natural killer cell; TGFβ1, transforming growth factor-β1; ECM, extracellular matrix; VEGF, vascular endothelial growth factor.

MSCs support hepatic restoration through multifaceted paracrine, immunomodulatory, antifibrotic, metabolic, and angiogenic mechanisms. MSCs coordinate multiple regenerative pathways within the hepatic microenvironment, orchestrating tissue repair through these mechanisms (Figure 1 and Table 3). The key mechanisms, including trophic and angiogenic factors, immunomodulation, antifibrotic and metabolic effects, and extracellular vesicle (EV)-mediated signaling, are summarized in Table 3. MSC effects do not occur strictly sequentially; rather, they represent overlapping processes with variable predominance depending on the phase of liver injury and repair. Consistent with this concept, immunomodulatory effects, such as suppression of inflammatory cytokines and modulation of innate immune cells, are often evident shortly after MSC administration [47,48,49]. Antifibrotic effects, including inhibition of hepatic stellate cell activation and modulation of intrahepatic B cells, become more prominent during tissue remodeling [50,51,52]. Over longer time frames, MSC-mediated metabolic and regenerative effects, such as amelioration of hepatocyte lipid load and promotion of fatty acid oxidation, together with favorable angiogenesis-associated vascular remodeling, have been reported to contribute to long-term hepatic restoration [53,54,55]. Detailed experimental evidence in preclinical models and therapeutic applications is summarized in Section 6, whereas Section 4 outlines MSC- and EV-based therapeutic strategies.

## 4. Cell-Based Versus Cell-Free Therapeutic Strategies

As described in Section 3, MSCs possess the ability to broadly reprogram the immune response, making them suitable for treating complex liver diseases. However, 2D–cultured MSCs exhibit increased expression of integrins such as β1, α5, and αVβ3, which leads to their entrapment in the lungs after systemic administration via intravenous injection [64]. In addition, an increased passage number reduces homing to the bone marrow and spleen, whereas prior irradiation of the recipient enhances MSC homing to these organs [65]. Long-term culture of MSCs increases senescence and aneuploidy [66]. Taken together, the clinical applicability of MSCs faces several limitations, including variable homing depending on recipient age and status, pulmonary first-pass entrapment after intravenous injection, and genetic instability.

MSC-EVs recapitulate many of the therapeutic effects of MSCs through the delivery of therapeutic RNA and proteins (summarized in Figure 1), without the risks associated with live cells [67]. They exhibit low immunogenicity, can be sterilized by filtration, and are amenable to long-term storage and dose standardization using particle or protein metrics [68,69]. These attributes simplify logistics and regulatory compliance, making EVs an attractive candidate for off-the-shelf therapies.

Nevertheless, the clinical application of EV-based therapies necessitates the development of methods for not only isolating EVs but also preserving their functional integrity while enabling large-scale production [70]. In addition, scalable methods for isolating EVs from nonvesicular extracellular particles, measures for ensuring batch consistency, and robust potency assays for characterization are still evolving [71,72]. Furthermore, unlike living MSCs, EVs cannot modulate their functions according to the environment in the body, so their therapeutic effects may be limited in certain situations [73].

Several factors, including MSC/EV source, priming or reconditioning, delivery route, and disease stage, critically influence therapeutic success and should be carefully considered when designing treatment strategies. Quantitative comparisons indicate that perinatal-derived MSCs, such as UC-MSCs or PL-MSCs, achieve 20–40% greater reduction in liver fibrosis markers and enhanced hepatocyte proliferation than adult tissue-derived MSCs, likely due to higher proliferative capacity and enriched secretion of regenerative factors [74,75,76]. Preconditioning or priming MSCs (e.g., hypoxia or inflammatory stimuli) can enhance their homing ability and boost secretion of anti-inflammatory cytokines and growth factors, further improving antifibrotic efficacy by 15–25% [77,78]. The delivery route also influences therapeutic outcomes. Intrahepatic or portal vein injection allows more direct targeting of the liver, whereas systemic IV infusion may result in off-target trapping [75,79]. Finally, the stage of liver disease is important. MSC therapies are generally more effective in early-stage fibrosis, when tissue damage is reversible, while advanced cirrhosis with permanent architectural changes shows less responsiveness to MSC therapy [76,80,81].

Therefore, a complementary paradigm has emerged, recommending the deployment of living MSCs when adaptive immunomodulation is paramount and EVs when standardized anti-inflammatory and antifibrotic effects are sufficient [82,83,84].

## 5. Delivery Methods and Biomaterial-Based Therapeutic Enhancement

### 5.1. Influence of Administration Route and Dosing on Therapeutic Efficacy

The administration route significantly influences biodistribution, therapeutic efficacy, and procedural risk, especially in therapies involving MSCs and EVs [85,86,87,88]. Common delivery methods include peripheral intravenous (IV) infusion, portal vein or hepatic artery injection, and localized intrahepatic delivery.

IV administration is the simplest and least invasive method, making it convenient and often preferred. However, IV-injected MSCs are initially trapped in the lungs and rapidly die, which limits hepatic targeting and motivates the development of strategies to enhance liver-specific biodistribution [85,89].

**Figure 2:**
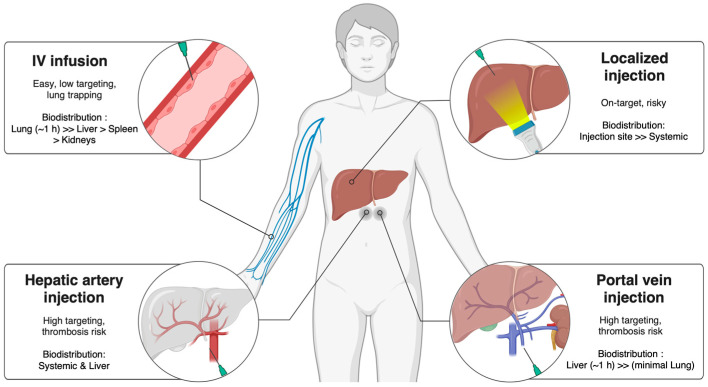
Delivery routes of MSCs and EVs in liver disease treatment. Biodistribution and therapeutic efficacy can be influenced by the route of administration, such as IV, portal vein, hepatic arterial, or localized injection. Figure created with Biorender (biorender.com).

By contrast, direct hepatic delivery via the portal vein or hepatic artery offers higher local (on-target) concentrations and improved engraftment [90]. However, these approaches require angiographic imaging guidance for catheterization of the portal vein or hepatic artery, as well as specialized interventional expertise [90]. They also carry higher risks of thrombosis and bleeding, which are particularly problematic in cirrhotic patients because of their often impaired hepatic vascular architecture, portal hypertension, coagulopathy, and fragile vessels and extensive collateral circulation [90,91,92,93]. Imaging-guided localized injections enable precise delivery to specific liver lesions or fibrotic areas, but this procedure requires high technical skill, careful planning, accurate imaging, precise needle placement, and risk management [94,95]. Furthermore, when the disease is widespread rather than focal, this approach becomes impractical or ineffective [94,95]. The delivery routes of MSCs and EVs in the treatment of liver disease are summarized in Figure 2.

Studies have employed a variety of dosing strategies depending on the disease model and indication.

In preclinical ALI models, MSC doses of 1–5 × 10^6^ cells/kg are commonly used, with intermediate doses showing maximal efficacy and higher doses producing little additional benefit [96,97,98]. In chronic liver injury (CLI) or fibrosis models, doses of 0.5–3 × 10^6^ cells/kg are typical, and repeated dosing frequently outperforms single administration, suggesting cumulative or maintenance effects [99,100,101,102]. MSC-derived EVs are generally administered at 10^9^–10^12^ particles/kg or 50–600 µg protein, with low doses showing some efficacy and intermediate doses yielding optimal therapeutic responses [103,104,105]. A detailed summary of indication-specific dose ranges and dose–response trends is provided in Table 4. These findings underscore the need for careful titration and pharmacodynamic monitoring to optimize therapeutic outcomes [96,97,98,99,100,101,102,103,104,105,106].

### 5.2. Role of Biomaterials in Enhancing Therapeutic Effects

Biomaterials such as hydrogels, scaffolds, and nanoparticles are being increasingly employed to augment the efficacy of MSC and EV therapies. These materials improve local retention, sustain therapeutic release, and provide supportive microenvironments conducive to tissue repair [107,108,109].

Hydrogels, which are composed of hydrophilic polymer networks that mimic the ECM, can encapsulate MSCs or EVs for localized delivery [110,111]. They retain therapeutic agents at the target site, protect them from immune clearance, and modulate paracrine signaling by providing a three-dimensional microenvironment [112,113,114,115]. Thermosensitive or injectable hydrogels facilitate minimally invasive administration with in situ gelation [116,117,118]. Preclinical models have demonstrated that hydrogel-based MSC delivery augments anti-inflammatory and antifibrotic effects in liver fibrosis [111,119].

Scaffolds provide mechanical support and biochemical cues that enhance cell survival, differentiation, and intercellular communication [120,121,122,123,124]. In the context of liver disease, bioengineered scaffolds, constructed from collagen, decellularized liver matrix, or synthetic polymers, promote hepatic tissue regeneration and fibrosis attenuation through favorable cell–matrix interactions [125,126,127,128,129]. Moreover, scaffolds can co-deliver MSCs and bioactive factors, offering a multifaceted therapeutic strategy [130,131,132,133].

**Figure 3:**
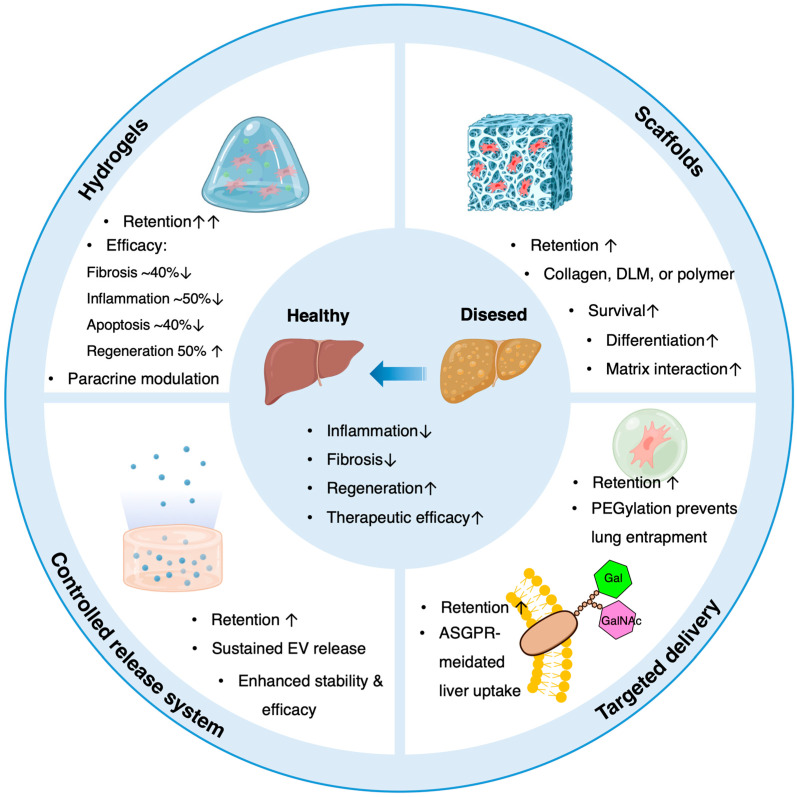
Biomaterial-assisted strategies for enhancing MSC and EV therapy in liver disease. Biomaterials such as hydrogels and scaffolds enhance retention, immune protection, and targeted release to improve therapeutic outcomes. PEGylation allows MSCs to bypass the lungs and enhances liver targeting, while exosomes with exposed Gal and GalNAc moieties are recognized by hepatocytes. Relative increases in MSC/EV retention and therapeutic efficacy are indicated. Precise numerical values are largely unavailable in the current literature; arrows and relative magnitude reflect reported trends. Graphics created with Biorender (https://www.biorender.com). DLM, decellularized liver scaffold; EV, extracellular vesicle; Gal, D-galactose; GalNAc, N-acetyl-D-galactosamine; MSC, mesenchymal stromal/stem cell; ASGPR, asialoglycoprotein receptor; ↑, up-regulation; ↓, down-regulation.

Controlled-release systems, including nanoparticles, liposomes, and hydrogel composites, enable sustained EV release, improving stability and extending the therapeutic window [111,134,135,136]. This approach reduces dosing frequency and enhances patient compliance, which is particularly important in chronic liver diseases requiring repeated administration [137,138]. Targeted delivery is further optimized by modifying the surface of carriers with ligands recognizing liver-specific receptors, such as asialoglycoprotein receptor, thereby enhancing on-target efficacy and minimizing off-target effects [139,140,141,142]. For instance, galactose or N-acetylgalactosamine residues displayed on modified MSC-EVs can specifically bind asialoglycoprotein receptor on hepatocytes, promoting receptor-mediated endocytosis and efficient intracellular uptake [142]. Strategies such as PEGylation of MSCs have also been shown to reduce nonspecific adhesion in the lung, facilitating delivery to target sites [141]. Figure 3 summarizes biomaterial-assisted strategies for enhancing MSC and EV therapy in liver disease.

## 6. Preclinical Evidence of MSC/EV Therapy Across Liver Injury Models

Factors affecting MSC/EV therapeutic success, including cell source, preconditioning, deliver route, and disease stage, are described in Section 4.

### 6.1. Acute Liver Injury Models

In rodent models of ALI, such as acetaminophen- or CCl_4_-induced hepatotoxicity, MSCs have consistently suppressed hepatocyte apoptosis and necrosis and inflammatory cytokine levels, while improving survival and liver function [143,144]. As summarized in Table 5, these effects are largely mediated through various anti-inflammatory cytokines and trophic factors [144]. In acetaminophen overdose, toxic metabolites such as N-acetyl-p-benzoquinone imine are generated in the liver, causing oxidative stress and mitochondrial damage in hepatocytes [59]. MSCs also suppress the expression of proinflammatory cytokines such as TNF-α and IL-1β, resulting in improved liver function and survival rates in rodent models [145].

CCl_4_ is metabolized by hepatic cytochrome P450 2E1 into free radicals, which cause lipid peroxidation and hepatocyte damage [143]. MSC administration provides anti-oxidant effects and regulates the inflammatory response to promote liver tissue regeneration (Table 4) [143]. Additionally, in the CCl_4_ model, MSCs and MSC-EVs inhibit the progression of fibrosis, indicating their potential role in preventing and reversing liver fibrosis [143,146,147].

### 6.2. Ischemia–Reperfusion and Partial Hepatectomy

MSCs have demonstrated significant efficacy in enhancing liver regeneration following ischemia–reperfusion injury and partial hepatectomy in rodent models and preclinical studies [148]. Ischemia–reperfusion injury, a common complication during liver surgery and transplantation, induces oxidative stress, inflammation, and hepatocyte apoptosis. These processes impair liver function and regeneration capacity. Administration of MSCs has been shown to accelerate hepatic mass restoration by promoting hepatocyte proliferation and survival [149]. This regenerative effect is mediated by the secretion of various growth factors and cytokines detailed in Table 5, which collectively stimulate angiogenesis, reduce inflammatory responses, and inhibit oxidative damage [150].

Furthermore, MSCs contribute to microvascular reconstruction by enhancing endothelial cell proliferation and stabilization of the hepatic sinusoidal network, which is crucial for restoring proper blood flow and oxygen delivery to the regenerating liver tissue [149]. By mitigating the generation of reactive oxygen species and downregulating proinflammatory cytokines such as TNF-α and IL-6, MSCs alleviate reperfusion-induced hepatic injury and postoperative liver dysfunction [149].

In partial hepatectomy models, MSC therapy has been associated with increased expression of proliferating cell nuclear antigen and other markers of cell cycle progression, indicating enhanced hepatocyte regeneration [148,150]. These effects improve overall liver function, as evidenced by normalized serum levels of liver enzymes (alanine aminotransferase, aspartate aminotransferase) and bilirubin [150]. Additionally, MSCs modulate the hepatic microenvironment by interacting with resident immune cells, such as Kupffer cells and HSCs, to create a milieu conducive to tissue repair and fibrosis prevention [148]. Together, these multifaceted mechanisms support the therapeutic potential of MSCs as adjunct treatments in liver surgery and transplantation to improve outcomes, reduce complications, and promote faster recovery.

### 6.3. Chronic Liver Fibrosis and Cirrhosis Models

Chronic liver fibrosis and cirrhosis are progressive conditions marked by excessive ECM deposition and distortion of liver architecture, leading to impaired function and portal hypertension. MSCs and MSC-EVs have demonstrated strong antifibrotic effects in rodent models of liver fibrosis induced by CCl_4_, bile duct ligation, or thioacetamide [76,151,152,153]. MSCs reduce collagen deposition by downregulating profibrogenic factors associated with HSC activation, which is the main driver of fibrosis (see Table 5 for molecular targets) [52,154,155]. Through paracrine signaling, MSCs inhibit HSC proliferation, decrease ECM synthesis, and promote ECM degradation by regulating MMPs and tissue inhibitors of metalloproteinases, aiding in liver structure restoration and functional recovery [151,155].

Long-term MSC treatment lowers portal pressure and improves survival in chronic injury models. MSCs also modulate inflammation by reducing the content of proinflammatory mediators, further limiting fibrosis [76,156].

MSC-EVs offer the promise of cell-free therapy, delivering antifibrotic microRNAs (e.g., miR-122, summarized in Table 5), which target fibrosis-related pathways [52,157,158]. EVs replicate many benefits of MSCs, including attenuating fibrosis and controlling inflammation, with the added advantages of lower immunogenicity and easier handling [76,151]. Overall, MSCs and MSC-EVs have demonstrated notable potential to halt and reverse chronic liver fibrosis and cirrhosis, supporting their development for clinical applications [76,151,152].

### 6.4. Combination Therapies in Preclinical Studies

Combining MSCs or EVs with antifibrotic agents (e.g., pirfenidone), antivirals, or metabolic modulators (e.g., peroxisome proliferator-activated receptor-γ agonists) has yielded additive or synergistic benefits in animal models [159,160,161]. Such combination regimens enhance ECM remodeling, restore hepatocyte function, and reduce inflammation more effectively than monotherapy [159,160]. These findings support the rationale for integrative strategies in future clinical trials.

The integrated therapeutic mechanisms and outcomes of MSCs/EVs in these various models are summarized in Table 5.

## 7. Clinical Landscape: Safety and Efficacy

### 7.1. Early-Phase Clinical Trials Using MSCs

Multiple early-phase clinical trials have investigated the safety and preliminary efficacy of autologous or allogeneic MSC therapy in patients with liver cirrhosis, acute-on-chronic liver failure (ACLF), and other forms of hepatic decompensation [162,163,164]. Across these studies, MSC infusion has demonstrated a favorable safety profile, with no major infusion-related adverse events or tumorigenic complications reported [162,163,164,165]. Preliminary efficacy indicators include improvements in liver function parameters such as serum albumin and bilirubin, prothrombin time, and Model for End-stage Liver Disease (MELD) and Child–Pugh scores [162,163]. Table 6 summarizes early-phase clinical studies of MSCs in liver disease, including disease indication, MSC source, route of administration, sample sizes, follow-up duration, key outcomes, and adverse events. In these early-phase, short-term trials, clinical benefit was often observed only in specific patient subgroups, such as those with less severe disease (lower MELD or Child-Pugh scores), preserved synthetic liver function (higher baseline albumin or prothrombin activity), HBV-related etiology, or lower baseline systemic inflammation [164,166,167,168]. The durability of response beyond 6–12 months remains uncertain, because these studies generally lacked long-term follow-up [164,168,169,170]. The apparent limited or inconsistent duration of efficacy in these short-term studies may reflect progressive liver injury, persistent inflammatory milieu, and/or limited engraftment or survival of infused MSCs in the hostile hepatic environment [166].

Importantly, separate long-term follow-up studies reported sustained benefits, including improved survival and liver function over several years [165,166,167,171]. Higher MSC doses (2 × 10^8^ cells) and repeated administration (weekly for 3 weeks) improved liver function, as evidenced by Child–Pugh and MELD scores, along with a reduction in the proportion of MX1^+^ monocytes. These observations suggest that dose, repeated administration, and patient characteristics may influence response. Larger controlled trials are needed to confirm these clinical findings [166].

**Table 6:** Clinical studies of MSCs in liver disease by indication and route.

Study Reference	Disease	MSC/EV Source	Route of Administration	Phase	Sample Size	Follow-Up Duration	Key Outcomes	Safety/ Adverse Events
Shi et al., 2012 [164]	ACLF (HBV)	UC-MSCs	IV	Phase I/II	24 MSC/ 19 control	48–72 weeks	Improved liver function (ALB ↑, ChE ↑, PTA ↑, PLT ↑, TBIL ↓, ALT ↓, AST ↓); Early immune modulation; Survival at 24–48 weeks	No serious adverse events; Transient fever in 2 patients
Schacher et al., 2021 [169]	ACLF Grades 2&3	BM-MSCs	IV	Phase I-II RCT	(4 MSC/5 control)	90 days	No significant survival benefit (25% vs. 20%); one MSC patient showed improved Child-Pugh & MELD scores	No infusion-related severe adverse events; unrelated mild adverse events (hypernatremia, ulcer)
Cui et al., 2025 [168]	ACLF (various etiologies)	Off-the-shelf UC-MSCs	IV	Phase I/II	~30–50	12–14 weeks	Preliminary improvement in liver function and survival; decreases in MELD and TBIL suggested	No serious adverse events reported in early data
Shi et al., 2021 [165]	DLC (HBV)	UC-MSCs	IV	RCT	108 MSC/ 111 control	~75 months	Improved liver function (ALB ↑, ChE ↑, PTA ↑, INR ↓, TBIL ↓, ALT ↓, AST ↓, MELD ↓); Improved ascites & edema; Long-term survival (13–75 months); Immune modulation	No serious adverse events; No increased HCC
Li et al., 2023 [172]	DLC (HBV)	UC-MSCs	IV	Retrospective cohort	36 SCT vs. 72 matched control	Up to ~92 months	Improved long-term survival (3-year: 83.3% vs. 61.8%; 5-yr:63.9% vs. 43.6%); No increase in HCC	No increase in malignancy; mild transient fever in 3 patients
Shi et al., 2025 [166]	DLC	UC-MSCs	IV	Phase Ia/Ib	6–24 per cohort	~28 days	Improved liver function (ALB ↑, MELD ↓, TB↓ ↓, INR ↓, Cr ↓); enhanced synthetic/coagulation capacity; dose-dependent immune modulation (MX1^+^ monocytes ↑).	No dose-limiting toxicity; no significant adverse events
Qin et al., 2025 [167]	DLC (HBV)	UC-MSCs	IV	Clinical trial (single-arm)	24 (3 dose groups)	24 weeks (+2-year survival)	ALB transient ↑ (d57/85), sustained PTA ↑ (d29–d169); IL-8 ↓; 6-month survival 100%; 2-year survival 66.7–100%	No serious adverse events reported; favorably safety
Kharaziha et al., 2009 [170]	Liver cirrhosis (various etiologies: HBV, HCV, alcoholic, cryptogenic)	BM-MSCs	IV or portal vein	Phase I/II	~20–30	6–12 months	Improved liver function (ALB ↑, MELD ↓, TB ↓, INR ↓, Cr ↓); enhanced synthetic/coagulation capacity	Well tolerated; no significant adverse events

ACLF, acute-on-chronic liver failure; ALB, albumin; AST, aspartate aminotransferase; ALT, alanine aminotransferase; BM-MSCs, bone marrow-derived MSCs; ChE, cholinesterase; Cr, creatinine; d, day; DLC, decompensated liver cirrhosis; HBV, hepatitis B virus; HCV, hepatitis C virus; IV, intravenous; MELD, Model for End-stage Liver Disease; MSCs, mesenchymal stromal/stem cells; PLT, platelet count; PTA, prothrombin activity; RCT, randomized controlled clinical trial; SCT, stem cell transplantation; TBIL, total bilirubin; UC-MSCs, umbilical cord blood–derived MSCs; We added explanation; up-regulation; ↓, down-regulation.

Among MSC sources, UC-MSCs have shown the most consistent improvements. Serum albumin increased by 5–15 g/L, prothrombin activity improved by 10–20%, and total bilirubin decreased by 20–35% in short- to long-term follow-up [164,166,167,168,169,172]. MELD scores decreased by 2–5 points and Child–Pugh scores improved by 1–3 points, with long-term survival benefits up to 75–92 months [171,172]. Mild transient fever was the most commonly reported adverse event, and dose-dependent immunomodulatory effects, including reductions in MX1^+^ monocytes and IL-8, were observed [166]. BM-MSCs also improved liver function, with albumin increases of ~5–10 g/L, MELD decreases of ~1–3 points, and total bilirubin reductions of ~15–25% [169,170]. However, survival benefits were less consistent, particularly in small RCTs with short follow-up. Both intravenous and portal vein administration were well tolerated, with no serious adverse events reported. Overall, UC-MSCs appear to provide more sustained functional and survival benefits than BM-MSCs, while AD-MSCs have very limited clinical data, and iMSCs have only been investigated preclinically.

### 7.2. EV-Based Clinical Studies: Emerging Data

EV-based therapies are in the early stages of clinical testing. Phase I trials have confirmed the feasibility and safety of MSC-EVs in liver and non-liver indications, such as graft-versus-host disease and inflammation related to coronavirus disease 2019 [76,173]. Ongoing studies aim to evaluate efficacy endpoints in liver diseases, but data remain limited [76]. The development of potency assays and standardized EV products will be critical for advancing this field [174].

### 7.3. Challenges in Translating Preclinical Success to Clinical Benefit

Despite the robust evidence supporting the efficacy of MSC-based therapies in preclinical liver injury models, translating these effects into consistent clinical benefits has proven challenging [76,162]. One major obstacle is patient heterogeneity; differences in disease etiology, including viral hepatitis, alcoholic liver disease, and MASH, as well as variations in fibrosis stage and comorbidities, complicate the reliable prediction of therapeutic responses [162,165]. This heterogeneity likely contributes to the variability in clinical outcomes observed across studies, highlighting the importance of stratified study designs.

Many clinical studies rely primarily on short-term biochemical markers to gauge efficacy, which may not accurately reflect meaningful long-term outcomes such as histological improvement or overall survival [165,175]. The durability of therapeutic effects also remains uncertain; most reported benefits tend to be transient, and long-term follow-up data are scarce [76,162,170], raising concerns about the clinical relevance of short-term improvements observed in current trials.

For example, meta-analysis (2013–2023) of BM-MSC therapy for cirrhosis or ACLF revealed trends toward better survival at 4 and 24 weeks. Nevertheless, no significant improvements were observed in ALT/AST (liver cell injury markers) or PT/INR (blood coagulation markers) [76,162,170]. Total bilirubin changes remained inconsistent, and the survival benefit at 24 weeks lacked statistical significance [76,162,170]. These results suggest that improvements in short-term laboratory markers may not fully capture the complex mechanisms by which MSC therapy could affect patient survival, highlighting the need for longer-term and more comprehensive clinical endpoints.

Inconsistencies in trial design, including variations in MSC sources, dosing regimens, and delivery methods, further limit comparability and may obscure the true therapeutic potential [165,175]. Differences in cell preparation can affect engraftment, paracrine signaling, and immunomodulatory capacity, which may explain why promising preclinical results are not consistently replicated in heterogeneous patient populations.

Overall, the currently reported limited clinical efficacy of MSC therapy can be attributed to patient heterogeneity, a lack of standardized dosing, variability in MSC sources and protocols, and the transient nature of therapeutic benefits. To address these challenges, future clinical trials should adopt stratified study designs that account for patient heterogeneity, implement standardized and clinically relevant endpoints, incorporate biomarker-guided monitoring strategies, and extend follow-up periods to capture hard outcomes such as transplant-free survival and improvements in quality of life [76,162,165,170,175].

### 7.4. Conflicting Preclinical and Clinical Evidence

Although MSC and MSC-EV therapies have shown promise, several preclinical and clinical studies report inconsistent or negative outcomes, highlighting that these therapies are not universally effective. In preclinical models, some studies observed poor MSC engraftment, variable EV potency, and, in rare cases, pro-tumorigenic effects [143,146,151]. Therapeutic responses also differed by disease phase and etiology, with chronic fibrosis or cirrhosis often responding less effectively than acute injury [143,151,153].

Clinically, many trials reported no significant improvement in liver injury markers such as ALT, AST, PT/INR, or total bilirubin, despite occasional trends toward short-term survival benefit [162,165,170,175]. Observed effects were often transient, and long-term follow-up data remain limited [162]. These findings collectively emphasize that MSC and MSC-EV therapies may provide benefits in certain contexts but are not consistently effective across all models, patient populations, or disease stages.

## 8. Manufacturing, Quality Control, and Safety Considerations

### 8.1. Good Manufacturing Practice–Compliant Manufacturing Processes

The transition of MSC and EV-based therapies from bench to bedside requires adherence to GMP standards [176,177]. This involves the use of closed, automated bioreactor systems to minimize contamination risk and ensure scalability [178,179]. Culture in xeno- and serum-free media is now standard practice because it eliminates animal-derived components that pose the risk of immunogenicity and pathogen transmission [176,179]. Given that prolonged culture can degrade the functional potency of cells, cell expansion protocols must also control for senescence and phenotype drift [176]. For EVs, scalable isolation methods such as tangential flow filtration and size-exclusion chromatography are being optimized to support clinical-grade production [176,179]. Cryopreservation protocols must preserve functionality post-thaw and enable batch tracking [176]. However, the reliance on GMP-compliant closed systems, specialized downstream processing, and advanced quality control substantially increases manufacturing costs and limits scalability to specialized facilities. Clinical-grade EV production should consider the recommendations from MISEV2023, which outline minimal criteria for EV characterization and reporting [180]. Practical barriers include scalability, maintaining consistent EV cargo, and adapting MISEV2023 guidelines into closed-system GMP bioreactors [76,181].

### 8.2. Quality Control and Potency Assays

Comprehensive quality control is essential to ensure the consistency and safety of MSC-based products across different production batches [182,183]. For MSCs, typical release criteria include confirming cell identity through flow cytometry by assessing the presence of characteristic surface markers such as CD73, CD90, and CD105, alongside the absence of hematopoietic markers such as CD45 [182,184,185]. Viability is another critical parameter, with acceptable levels generally exceeding 70% [184]. Sterility testing is conducted to exclude contamination from endotoxins, mycoplasma, and other microbial agents [182]. Genomic stability is assessed using techniques such as karyotyping and single nucleotide polymorphism arrays to detect chromosomal abnormalities [182]. Potency assays should focus on functional activities directly correlating with liver outcomes, including immunosuppressive capacity, inhibition of HSC activation, and modulation of macrophage polarization [181,186]. Additionally, potency assays are performed to evaluate functional activity relevant to therapeutic goals, including immunosuppressive capacity, macrophage polarization, and inhibition of HSC activation [183]. However, despite considerable advancements in technology, standardized approaches for producing and quantifying exosomes remain underdeveloped, including methods for profiling microRNA and protein cargo and assessing their therapeutic efficacy.

This bottleneck limits the translation of exosomes into clinical practice [187,188]. Comparative evaluation of isolation methods-such as ultracentrifugation, size-exclusion chromatography, and tangential flow filtration-should consider preservation of critical therapeutic cargo (e.g., miR-122, TGF-β1) and correlation with potency assays [174,189]. Functional assays for EVs often focus either on their ability to suppress pro-fibrotic and proinflammatory mediators, such as TGF-β and IL-6, or to inhibit HSC activation [187]. Moreover, integrating MISEV2023 recommendations can improve standardization, but practical barriers remain, including batch-to-batch variability, scalability challenges, and compatibility with closed-system GMP manufacturing [181]. However, to demonstrate that the product is consistent and truly relevant for disease treatment, validated assays that correlate with clinical efficacy are also required [183,188].

### 8.3. EV Characterization and Standardization

EVs present unique challenges in the form of their heterogeneity and the variability of their cargo. Current efforts are focused on standardizing isolation protocols and developing potency assays that are rooted in the mechanisms of action of the EVs and predictive of their therapeutic outcomes. Clinical-grade EV production should consider the recommendations from MISEV2023, which outline minimal criteria for EV characterization and reporting [181]. Practical barriers include scalability, maintaining consistent EV cargo, and adapting MISEV2023 guidelines into closed-system GMP bioreactors [181].

According to the guidelines of the International Society for EVs, detailed characterization is essential and includes analysis of size distribution and morphology using techniques such as transmission electron microscopy and nanoparticle tracking analysis; identification of surface markers such as CD63, CD81, and CD9; and profiling of RNA and protein cargo through methods such as quantitative PCR and mass spectrometry. Functional assays, including T-cell suppression, wound healing tests, and suppression of pro-fibrotic/proinflammatory mediators such as TGF-β1 and IL-6, are also recommended to link EV activity to liver therapeutic outcomes [174,189].

Furthermore, batch tracking, digital traceability, and stability testing under stress conditions are increasingly being integrated into advanced EV manufacturing pipelines to ensure quality and consistency [180]. In addition, EVs should be evaluated for reproducibility of cargo profiles and correlation with potency assays to meet clinical-grade manufacturing requirements [186,189]. Taken together, the development of clinical-grade EVs requires harmonization of potency assays, standardized isolation methods, and adherence to MISEV2023 characterization guidelines. To summarize the key aspects, clinical relevance, and practical barriers associated with implementing these recommendations, we provide an overview in Table 7.

## 9. Safety Concerns and Risk Mitigation

MSCs have a generally favorable safety profile, as demonstrated through extensive preclinical studies and clinical trials. However, several specific risks require careful management. Infusion reactions, including fever or chills, are typically mild and present shortly after infusion [190,191]. However, thromboembolic complications are among the most serious adverse events associated with MSC therapies administered via IV infusion. High cell doses or large infusion volumes can increase the risk of coagulation disorders or thromboembolic events, potentially owing to the expression of tissue factor, especially in cirrhotic patients who already have baseline coagulopathy [192]. The use of anticoagulation protocols or filtration systems may help mitigate this risk. Although uncommon, MSC therapy is also associated with the risk of tumor formation, which may result from genetic and epigenetic changes during in vitro expansion or modulation of the microenvironment in ways that promote tumor growth [193]. Consequently, stringent genomic stability assessments are warranted, and patients with active malignancies should be excluded from therapy [194]. Additionally, immunogenicity is an important consideration, especially for allogeneic MSCs, as repeated administrations may provoke immune responses that reduce therapeutic efficacy or lead to sensitization [195,196]. Table 8 summarizes the reported adverse events associated with MSC-based therapies in liver cirrhosis and acute-on-chronic liver failure, including the incidence and severity of common and serious adverse events. In comparison, EVs lack proliferative capacity and generally pose a lower immunogenic risk. Nevertheless, their long-term biodistribution, potential off-target effects, and immunomodulatory profiles require continued investigation to fully establish their safety [195,197,198].

## 10. Regulatory and Ethical Aspects

### 10.1. Regulatory Classification of MSCs and EVs

MSCs are regulated as advanced therapy medicinal products in the European Union and as biologics or Human Cells, Tissues, and Cellular and Tissue-Based Products in the United States, depending on the extent of their manipulation and intended clinical application [199,200,201]. EVs, however, occupy a less clearly defined regulatory space, often being classified as biologically derived products or novel biologics with regulatory approaches varying across regions [202,203,204]. In the European Union, the European Medicines Agency increasingly recognizes EVs under the advanced therapy medicinal products framework, which necessitates adherence to full GMP standards, comprehensive preclinical data, and clinical trial evaluation [199,201,204]. In the United States, the Food and Drug Administration regulates EVs under the Investigational New Drug application process, with guidance documents emphasizing thorough product characterization, demonstration of potency, and risk mitigation strategies [203,204]. Meanwhile, regulatory frameworks in Asia vary by country; for example, Japan offers expedited regulatory pathways for regenerative medicine products under the “Sakigake” designation, facilitating faster clinical development and approval [205,206].

### 10.2. Ethical Sourcing and Donor Eligibility

Ethical procurement of MSC sources, particularly perinatal tissues, requires informed consent, transparency, and adherence to donor eligibility criteria [207,208]. Donors must undergo rigorous infectious disease screening and be free from transmissible conditions [209]. Autologous therapies pose fewer ethical dilemmas but offer lower scalability [210]. For allogeneic sources, equitable tissue access, benefit-sharing, and avoidance of commercial exploitation are important ethical pillars [211].

### 10.3. Post-Market Surveillance, Cost, and Scalability Considerations

Post-marketing surveillance systems, such as pharmacovigilance and adverse event tracking, are essential to ensure ongoing safety and effectiveness [212]. Real-world data from registries can inform treatment algorithms and risk management strategies [212]. Cost-effectiveness and scalability play a decisive role in reimbursement and adoption [213], and MSC- or EV-based therapies must demonstrate meaningful improvements over the standard of care, including reductions in liver-related complications, delays in transplantation, or improvements in quality-adjusted life years [213].

Evidence suggests that EV production may be theoretically more cost-efficient than MSC expansion due to shorter culture times, reduced dependence on expensive growth factors during specialized collection phases, and simpler long-term storage (e.g., lyophilization) [214,215,216,217]. Techno-economic modeling studies based on GMP-like co-production platforms have estimated substantially lower per-dose manufacturing costs for MSC-EVs compared with MSCs (approximately €166–3082 versus €965–42,673, respectively) [218]. However, these best-case scenarios rely on shared upstream manufacturing and may not apply to standalone EV production.

Under current GMP conditions, EV manufacturing remains resource-intensive due to complex downstream processing (DSP), including isolation, purification, and characterization requirements, resulting in high GMP-related manufacturing costs [219,220]. While MSC-based therapies also incur substantial costs related to large-scale cell expansion, cryopreservation, and batch release testing, their manufacturing processes are relatively well-established [221]. Additionally, the lack of standardized EV production and potency assays, combined with low recovery yields during high-purity filtration, and the need for specialized infrastructure, represent key barriers to widespread clinical translation [222].

## 11. Biomarkers and Patient Stratification

### 11.1. Predictive and Pharmacodynamic Biomarkers

Reliable biomarkers are crucial for selecting patients who are most likely to respond to MSC-based therapies and for monitoring treatment progress, which in turn can inform dosing strategies. Multiple potential biomarkers have been identified across genomic, proteomic, and metabolomic landscapes. The levels of cytokines, including IL-6, TNF-α, and IL-10, may reflect important immunologic shifts associated with a therapeutic response [223,224]. For example, IL-6 has been shown to have sensitivity of 80% and specificity of 85% in predicting response to MSC-based therapies in patients with liver fibrosis [225]. Markers related to ECM remodeling, such as hyaluronic acid, tissue inhibitor of metalloproteinases-1, and procollagen III N-terminal peptide, serve as indicators of fibrosis activity and regression [226,227,228]. Hyaluronic acid levels in particular have shown sensitivity of 75% and specificity of 80% in detecting liver fibrosis progression [229]. Additionally, EV-associated microRNAs, such as miR-122 and miR-19, have been shown to be correlated with hepatic regeneration and antifibrotic responses, with miR-122 demonstrating a cut-off value of 3.2 ng/mL for indicating significant liver injury in patients with MASLD [230,231]. Continuous monitoring of these biomarkers during treatment helps assess therapeutic effectiveness and enables personalized adjustments, thereby enhancing overall efficacy and patient outcomes.

### 11.2. Imaging Biomarkers and Noninvasive Endpoints

Advanced imaging techniques provide valuable noninvasive alternatives to traditional liver biopsy for assessing disease status and treatment response, while serum markers provide convenient and cost-effective tools for initial screening [225]. Transient elastography, conducted using the FibroScan device, measures liver stiffness and offers a rapid, patient-friendly method for evaluating liver fibrosis. This method can be performed in both outpatient and inpatient settings and provides immediate results [232]. For example, FibroScan has a cut-off value of 7.5 kPa for identifying significant liver fibrosis (F2 or higher), with a sensitivity of 85% and specificity of 90% [232]. However, measurement failure occurs in approximately 5% of the cases, primarily in obese patients, and the performance of this method in other liver diseases has not been fully validated yet [232]. Magnetic resonance imaging–based techniques such as T1 mapping (including iron-corrected cT1) and magnetic resonance elastography provide quantitative imaging biomarkers that reflect liver inflammation and fibrosis in patients with nonalcoholic fatty liver disease/MASH [233]. T1 mapping has shown sensitivity of 70% and specificity of 75% in detecting liver fibrosis in MASLD patients, though its diagnostic performance for MASH remains modest, and further validation is needed before routine clinical deployment [233]. Combining these imaging modalities with serum or plasma biomarkers can enhance the sensitivity and specificity of disease assessment in clinical trials, enabling more precise monitoring of therapeutic effects and disease progression.

### 11.3. Stratified Medicine Approaches

Etiology-specific responses to MSC-based therapies are gaining increasing recognition, highlighting the need for tailored treatment approaches [125,229]. Patients with MASH-related cirrhosis may derive greater benefit from interventions focused on anti-inflammatory effects and metabolic reprogramming [234,235,236]. By contrast, patients with alcoholic liver disease require careful consideration of treatment timing, particularly in relation to sustained abstinence and assessment of immune status [237,238]. Strategies that promote immune reconstitution or synergize with antiviral therapies may be particularly relevant for patients with hepatitis B virus– or hepatitis C virus–related fibrosis [239,240]. Incorporating patient stratification based on fibrosis stage, severity of portal hypertension, and immune biomarkers holds promise for improving response rates and enhancing the efficiency of clinical trials by targeting therapies to those most likely to benefit from them [241,242,243]. Furthermore, early shifts in macrophage polarization indices, such as M1/M2 ratio and cytokine secretion profiles, and HSC activation markers, such as α-smooth muscle actin expression and collagen production, could provide additional insight into therapeutic effects [171,244,245,246]. This integrated approach lays the foundation for optimizing MSC-based interventions and personalizing treatment strategies for liver disease.

## 12. Future Directions

MSCs exhibit variable characteristics depending on their tissue of origin and culture conditions, with potential risks of senescence and tumorigenesis. Therefore, to utilize MSCs as therapeutics, optimization of GMP-compliant culture conditions is essential [18,19,20,45,46]. Additionally, the establishment of standardized clinical protocols for administration routes, dosages, and integrated evaluation metrics would facilitate robust meta-analyses and informed regulatory decision-making. Although the effects of MSCs have been reported in various liver disease models, comparative studies conducted under identical conditions remain scarce, causing uncertainty regarding the optimal MSC origin and culture conditions [165,175]. Moreover, to establish accurate clinical indices and assess real clinical impact, long-term follow-up observations must be conducted over 6–12 months and randomized trials must be conducted in multiple organizations [76,162,165,170,175].

In the case of MSC-EVs, the key issues that need to be addressed are those related to large-scale production, purity, batch-to-batch consistency, preservation of functionality, and design of robust assays for safety evaluation [178,247,248]. In addition, through optimization of dosing and continuous development of biomaterials, off-target effects can be minimized, and stability and reproducibility can be enhanced [249].

Further studies on markers predicting liver disease progression and treatment response are needed to ascertain whether the therapeutic effect of antifibrotic and antiviral agents, metabolic interventions, and combination MSC/EV therapy could interact with each other additively. Single-cell analysis and spatial omics of human liver tissues can help elucidate the underlying mechanisms and select patients who are likely to respond to the therapy [151,250,251,252,253].

## 13. Literature Search Strategy

A systematic literature search was conducted using PubMed, covering publications from 1999 to 2025 to capture all relevant studies on MSCs and EVs in liver regeneration. To better reflect recent trends and advancements, particular attention was given to studies published between 2015 and 2025. Additionally, Google Scholar was searched to identify supplementary studies not indexed in the main databases.

The search terms included “mesenchymal stem cells”, “MSC”, “extracellular vesicles”, “EV”, “exosome”, “paracrine signaling”, “secretome”, “liver regeneration”, “hepatic injury”, “liver disease”, “liver fibrosis”, “liver inflammation”, “hepatic stellate cells”, “macrophage polarization”, “antifibrotic”, “immunomodulation”, “angiogenesis”, “scaffold”, “hydrogel”, “supporting material” “cell-free therapy”, “preclinical model”, and “clinical trial”.

Studies were included if they involved MSCs or EVs in preclinical or clinical models of liver injury, and excluded if they were unrelated to liver therapy, non-English, or lacked sufficient data.

## 14. Conclusions

MSCs and their EVs offer significant promise in regenerative hepatology, with potential to restore liver function and alleviate the need for transplantation. While preclinical and early clinical studies are promising, challenges remain in standardizing protocols, validating potency, and addressing regulatory issues. Advancements in biomaterials, EV engineering, and precision medicine provide a path toward scalable, safe, and effective therapies. This review integrates the latest evidence on MSC/EV biology, manufacturing, and regulatory considerations. Future research should focus on optimizing manufacturing processes, validating potency assays, and incorporating personalized medicine strategies. A proposed timeline includes short-term (1–2 years) efforts for GMP-compliant protocols, mid-term (3–5 years) multicenter trials, and long-term (5–10 years) broader clinical implementation.

## Figures and Tables

**Table 1:** Source dependent features of MSCs for hepatic applications.

Feature	BM-MSCs	AD-MSCs	Perinatal MSCs	iMSCs
Tissue Origin	Bone marrow	Adipose tissue	Wharton’s jelly, umbilical cord blood, placenta	iPSC-derived
Proliferation Capacity	Low (0.001–0.01% of nucleated cells)	High	Very high	Unlimited
Extraction Method	Invasive aspiration technique	Minimally invasive liposuction	Noninvasive collection from perinatal tissues	Complex manufacturing process
Immunologic Properties	Strong immunomodulatory effects	Immunomodulatory	Lower immunogenicity, strong anti-inflammatory and antifibrotic effects	Similar to native MSCs
Functional Characteristics	Strong osteogenic potential, immunomodulation, extensive clinical research	Efficient proliferation, potent proregenerative secretome, high cell yield suitable for scalable manufacturing	Retain primitive phenotype; elevated stemness markers such as OCT4 and SOX2; off-the-shelf allogeneic use	Resembling native MSCs in surface markers and functional characteristics; genomic instability, tumorigenic risk, complex manufacturing

BM-MSCs, bone marrow-derived mesenchymal stromal/stem cells; AD-MSCs, adipose-derived mesenchymal stromal/stem cells; iPSC, induced pluripotent stem cell; iMSCs, iPSC-derived mesenchymal stromal/stem cells.

**Table 2:** Influence of culture conditions on MSC potency and function.

Culture Variable	Condition	Key Molecular Changes	Functional Effects Relevant to Liver Disease
Oxygen tension	Hypoxia (1–5% O_2_)	HIF-1α/HIF-2α ↑ VEGF, HGF, bFGF ↑ enhanced histone acetylation	Enhanced self-renewal, angiogenesis, hepatoprotection, preserved differentiation capacity
Inflammatory preconditioning	IFN-γ, TNF-α	COX-2, PGE2, IDO, PD-L1 ↑ EVs enrichment of CD73, CD5L	Enhanced immunosuppression, macrophage M2 polarization, suppression of alloimmune responses
Culture dimensionality	3D spheroids	Mild hypoxia; IL-10, PGE2 ↑ HGF, SDF-1, MMPs ↑ proangiogenic miRNAs ↑	Stronger anti-inflammatory, antifibrotic, hepatoprotective and proangiogenic effects
Serum conditions	Xeno-/serum-free	Reduced xenogeneic proteins; improved expansion consistency	Improved GMP compliance, reduced immunogenicity, enhanced clinical safety

bFGF, basic fibroblast growth factor; COX-2, cyclooxygenase-2; EVs, extracellular vesicles; HGF, hepatocyte growth factor; HIF, hypoxia-inducible factor; IDO, indoleamine 2,3-dioxygenase; IL-10, interleukin-10; MMPs, matrix metalloproteinases; MSCs, mesenchymal stromal/stem cells; PD-L1, programmed death-ligand 1; PGE2, prostaglandin E2; SDF-1, stromal cell-derived factor-1; VEGF, vascular endothelial growth factor; GMP, Good Manufacturing Practice; ↑, up-regulation.

**Table 3:** MSC and EV dosing for liver disease: indication, dose range, administration rout, and dose–response trend.

Mechanism	Key Factors	Main Function	References
Paracrine/Trophic	HGF, VEGF, IGF-1, IL-6	Promote hepatocyte proliferation, inhibit apoptosis, enhance tissue perfusion	[56,57,58]
Immunologic	Reduction in CD8^+^ T cells, NK cells, B cells; M2 macrophage polarization; Prostaglandins inducing regulatory dendritic cells	Suppress immune response, promote anti-inflammatory environment, enhance tissue repair	[47,48,49,59]
Atifibrotic	miR-378c (exosomal), TGF-β1 inhibition	Inhibit hepatic stellate cell activation and fibrosis	[50,51]
Metabolic	Mitochondrial transfer via tunneling nanotubes	Restore hepatic oxidative capacity and lipid metabolism	[54]
Angiogenic	ANG-1, ANG-2, VEGF	Enhance angiogenesis and oxygen delivery during liver regeneration	[53]
EV-mediated/Paracrine	MicroRNAs (miR-19b, miR-122, miR-124, miR-182-5p, miR-148a), Proteins (DDX5, CAMKK1)	Modulate recipient cell behavior, restore hepatic function without cellular engraftment	[51,52,55,60,61,62,63]

ANG, angiopoietin; HGF, hepatocyte growth factor; IGF-1, insulin-like growth factor-1; IL-6, interleukin-6; miR, microRNA; NK, natural killer; VEGF, vascular endothelial growth factor.

**Table 4:** Key Mechanisms of MSC-Mediated Liver Regeneration.

Liver Injury Model	Therapy	Dose Range	Administration Route	Dose–Response	References
ALI (mouse/rat)	MSCs	1–5 × 10^6^ cells/kg	IV	1–2 × 10^6^: low effect; 2–4 × 10^6^: optimal/moderate effect; >4 × 10^6^: plateau or slight decrease in efficacy	[97,101]
CLI/fibrosis (mouse/rat)	MSCs	0.5–3 × 10^6^ cells/kg (or 1–20 × 10^7^ total cells)	IV, IP	0.5–1 × 10^6^: low effect; 1–3 × 10^6^: moderate effect; >3 × 10^6^: no further improvement	[101,105]
Decompensated cirrhosis (human, early clinical)	MSCs	5 × 10^7^–2 × 10^8^ cells/infusion	IV	5–10 × 10^7^: low effect; 1–2 × 10^8^: moderate/optimal; higher doses: no additional benefit observed	[104]
Liver injury (mouse/rat)	MSC-EVs	10^9^–10^12^ particles/kg or 50–600 µg protein	IV	10^9^–3 × 10^10^ particles (or 50–150 µg): low effect; 3 × 10^10^–1 × 10^11^ particles (150–300 µg): moderate effect; >1 × 10^11^ particles (300–600 µg): plateau	[102,106]
Ischemic liver/ stroke models	MSC-EVs	30–250 µg protein	IV	30–100 µg: low effect; 100–250 µg: moderate/optimal; repeated dosing may further enhance effect	[103,106]

ALI, acute liver injury; CLI, chronic liver injury; MSCs, mesenchymal stem cells; EVs, extracellular vesicles; IV, intravenous; IP, intraperitoneal; MSC-EVs, MSC-derived extracellular vesicles.

**Table 5:** Therapeutic effects and molecular mechanisms of MSCs and MSC-EV across preclinical liver injury models.

Liver Injury Model	Major Effects of MSC and MSC-EVs	Molecular Mechanisms	Key Features and Outcomes
ALI (APAP, CCl_4_, D-Gal)	Hepatocyte protection, improved survival, reduced inflammation	IL-10, HGF, PGE2, IGF-1 ↑; Iron deposition ↓ (via hepcidin/ferroportin 1); M2 macrophage polarization (via IL-4/Wnt-3a)	Rapid hepatoprotection; Attenuates necrosis and apoptosis; Restoration of iron homeostasis
Immune-mediated injury (ConA, *P. acnes*)	Suppression of systemic and local immune responses	CD8+ T cells, NK cells, B cells ↓; Regulatory DCs ↑ (via PGE2/EP4); Inhibition of S1P receptor 5 signaling	Mitigates fulminant hepatic failure; Regulates adaptive and innate immune cell trafficking
Ischemia–reperfusion (I/R)	Attenuated inflammation, improved liver function	ROS production ↓; Angiopoietin 1, Angiopoietin 2, VEGF ↑; TNF-α, IL-6 ↓	Protection against surgery- and transplant-related injury; Sinusoidal network stabilization
Partial hepatectomy	Accelerated liver regeneration, restored liver mass	HGF, VEGF ↑; Activation of PCNA and cell-cycle regulators	Promotes hepatocyte proliferation and microvascular reconstruction
Chronic fibrosis and cirrhosis	Antifibrotic effects, ECM remodeling, reduced portal pressure, improved survival	TGF-β1, α-SMA, intrahepatic B cells ↓; MMPs ↑; TIMPs ↓; miR-122, miR-19b, miR-378c (delivery via EVs)	Partial reversal of fibrosis; Inhibits HSC activation; EV therapy offers lower immunogenicity
Metabolic injury (MASH)	Recovery from metabolic impairment	Mitochondrial transfer via Tunneling Nanotubes (TNTs)	Lipid breakdown restoration; Improved oxidative capacity

ALI, acute liver injury; α-SMA, alpha-smooth muscle actin; ECM, extracellular matrix; EVs, extracellular vesicles; HGF, hepatocyte growth factor; HSC, hepatic stellate cell; IL-10, interleukin-10; IL-6, interleukin-6; miR, microRNA; MMPs, matrix metalloproteinases; PCNA, proliferating cell nuclear antigen; ROS, reactive oxygen species; TGF-β1, transforming growth factor-beta 1; TIMPs, tissue inhibitors of metalloproteinases; TNF-α, tumor necrosis factor-alpha; VEGF, vascular endothelial growth factor; ↑, up-regulation; ↓, down-regulation.

**Table 7:** Potency assays, isolation strategies, and practical barriers to implementing MISEV2023 in clinical-grade EV manufacturing.

Domain	Key Aspect	Clinical Relevance to Liver Disease	Practical Barriers (MISEV2023 Context)	References
Potency assay	HSC activation (α-SMA, collagen I)	Correlates with fibrosis regression	Lack of standardized assay thresholds	[186]
Potency assay	Macrophage M1/M2 polarization	Reflects anti-inflammatory response	Inter-assay variability	[186]
EV cargo	miR-122, miR-19b	Hepatocyte regeneration, antifibrotic signaling	Cargo heterogeneity across batches	[186]
Isolation	Ultracentrifugation	Widely used research method	Cargo loss, poor GMP scalability	[181]
Isolation	SEC	Better purity & cargo preservation	Low yield, scale limitation	[181]
Isolation	TFF	GMP-compatible, scalable	Requires process optimization	[181]
MISEV2023	Characterization requirements	Ensures rigor & reproducibility	Cost, time, equipment burden	[180,181]

α-SMA, alpha-smooth muscle actin; EV, extracellular vesicle; GMP, Good Manufacturing Practice; HSC, hepatic stellate cell; MISEV, Minimal Information for Studies of Extracellular Vesicles; SEC, size-exclusion chromatography; TFF, tangential flow filtration.

**Table 8:** Reported adverse events associated with MSC-based therapies in liver cirrhosis and acute-on-chronic liver failure.

Cell Source	Disease	Route	Adverse Event	Reported Incidence	Severity	References
BM-MSC, UC-MSC	Liver cirrhosis/ACLF	IV	Infusion-related reactions (fever, chills)	~5–20%	Mostly mild, transient, self-limiting	[162,163,164]
BM-MSC, UC-MSC	Liver cirrhosis/ACLF	IV	Thromboembolic/ coagulation events	<5%	Rare; higher risk in advanced cirrhosis; no consistent increase vs. controls	[162,163,193]
UC-MSC (dose escalation)	Decompensated cirrhosis	IV	Dose-limiting toxicity	Not observed	No DLT across dose cohorts	[166]
UC-MSC (off-the-shelf)	ACLF	IV	Serious adverse events	Comparable to control	Mostly disease-related; not MSC-attributable	[168]
BM-MSC	Decompensated cirrhosis	IV/ intrahepatic	Infection	No increase vs. control	No treatment-related infections reported	[165,169]
BM-MSC, UC-MSC	Cirrhosis/ACLF	IV	Tumorigenicity	Not reported	No de novo malignancies during follow-up	[162,163,165]
UC-MSC	HBV-associated cirrhosis	IV	Immunogenicity	Rare/subclinical	No clinically significant alloimmune reactions	[167,172]

MSC, mesenchymal stromal cells; BM-MSC, bone marrow-derived mesenchymal stromal cells; UC-MSC, umbilical cord-derived mesenchymal stromal cells; ACLF, acute-on-chronic liver failure; IV, intravenous; DLT, dose-limiting toxicity; HBV, hepatitis B virus.

## Data Availability

No new data were created or analyzed in this study. Data sharing is not applicable to this article.

## Abbreviations

The following abbreviations are used in this manuscript:

ACLF	Acute-on-chronic liver failure
AD-MSC	Adipose-derived MSCs
ALF	Acute liver failure
ALI	Acute liver injury
ALT	Alanine aminotransferase
APAP	Acetaminophen
ASGPR	Asialoglycoprotein receptor
AST	Aspartate aminotransferase
BM-MSC	Bone marrow-derived MSC
CCl_4_	Carbon tetrachloride
CLI	Chronic liver injury
ECM	Extracelluar matrix
EV	Extracellular vesicle
FHF	Fulminant hepatic failure
HBV	Hepatitis B virus
HCV	Hepatitis C virus
HGF	Hepatocyte growth factor
HIRI	Hepatic ischemia–reperfusion injury
HSC	Hepatic stellate cell
IL-6	Interleukin-6
IL-10	Interleukin-10
iMSC	iPSC-derived MSC
iPSC	Induced pluripotent stem cell
I/R injury	Ischemia–reperfusion injury
INR	International normalized ratio
IV	Intravenous
LPS	Lipopolysaccharide
MASH	Metabolic dysfunction-associated steatohepatitis
MASLD	Metabolic dysfunction-associated steatotic liver disease
MELD	Model for End-stage Liver Disease
MMP	Metalloproteinase
MSC	Mesenchymal stromal/stem cell
NK cell	Natural killer cell
Data	Data
PCNA	Proliferating cell nuclear antigen
PGE2	Prostaglandin E2
PL-MSC	Placenta-derived MSC
PT	Prothrombin time
ROS	Reactive oxygen species
SDF-1	Stromal cell-derived factor-1
Th1	T helper 1 cell
TIMP	Tissue inhibitor of Metalloproteinases
TIMP-1	Tissue inhibitor of Metalloproteinases-1
TNF-α	Tumor necrosis factor-alpha
TNTs	Tunneling nanotubes
UC-MSC	Umbilical cord blood-derived MSC
VEGF	Vascular endothelial growth factor
WJ-MSC	Wharton’s jelly-derived MSC
